# Association between Type-D Personality and Affective (Anxiety, Depression, Post-traumatic Stress) Symptoms and Maladaptive Coping in Breast Cancer Patients: A Longitudinal Study

**DOI:** 10.2174/1745017902117010271

**Published:** 2021-12-31

**Authors:** Luigi Grassi, Rosangela Caruso, Martino Belvederi Murri, Richard Fielding, Wendy Lam, Silvana Sabato, Silvia De Padova, Maria Giulia Nanni, Tatiana Bertelli, Laura Palagini, Luigi Zerbinati

**Affiliations:** 1 Department of Neuroscience and Rehabilitation, Institute of Psychiatry, University of Ferrara, Ferrara, Italy; 2 University Hospital Psychiatry Unit, University S. Anna Hospital and Local Health Trust, Ferrara, Italy; 3 Centre for Psycho-Oncological Research and Training, School of Public Health, The University of Hong Kong, Hong Kong School of Public Health, The University of Hong Kong, Pok Fu Lam, Hong Kong; Hong Kong Special Administrative Region, People's Republic of China; 4 Psycho-Oncology Unit, IRCCS Istituto Romagnolo per lo Studio dei Tumori (IRST) “Dino Amadori”, Meldola, Italy

**Keywords:** Type D personality, Breast cancer, Depression, Post-traumatic stress, Anxiety, Maladaptive coping

## Abstract

**Background:**

Type-D (distressed) personality has not been prospectively explored for its association with psychosocial distress symptoms in breast cancer patients.

**Objective:**

The objective of the study was to test the hypothesis that Type-D personality can be associated with psychosocial distress variables in cancer over a 2-point period (6 month-follow-up).

**Aims:**

The aim of the study was to analyze the role of Type-D personality in relation to anxiety, depression, post-traumatic stress symptoms, general distress, and maladaptive coping among cancer patients.

**Methods:**

145 breast cancer patients were assessed within 6 months from diagnosis (T0) and again 6 months later (T1). The Type-D personality Scale, the Hospital Anxiety and Depression Scale, Depression subscale (HAD-D), the Brief Symptom Inventory (BSI-18) Anxiety subscale, the Distress Thermometer (DT), the Post-traumatic Symptoms (PTS) Impact of Event Scale (IES), and the Mini Mental Adjustment to Cancer (Mini-MAC) Anxious Preoccupation and Hopelessness scales were individually administered at T0 and T1.

**Results:**

One-quarter of cancer patients met the criteria for Type-D personality, which was stable over the follow-up time. The two main constructs of Type-D personality, namely social inhibition (SI) and negative affectivity (NA), were related to anxiety, depression, PTS, BSI-general distress and maladaptive coping (Mini-MAC anxious preoccupation and hopelessness). In regression analysis, Type-D SI was the most significant factor associated with the above-mentioned psychosocial variables, both at T0 and T1.

**Conclusion:**

Likewise other medical disorders (especially cardiology), Type-D personality has been confirmed to be a construct significantly related to psychosocial distress conditions and maladaptive coping that are usually part of assessment and intervention in cancer care. More attention to personality issues is important in oncology.

## 1 INTRODUCTION

Studies focused on personality in cancer have mainly explored a specific personality style in cancer onset, a so-called “Type-C” cancer-prone personality [1-4]. Type-C has been defined to be characterized as a personal style comprising emotional non-expressiveness (or emotional suppression, especially anger), conflict avoidance, fear of social non-acceptance and need for approval from others, and a series of behaviors (*e.g*., submissiveness, pathological kindness and agreeableness, cooperativeness, excessive patience) [5-8]. Type-C attempts to replicate for cancer the Type-A hostile and impatient cardiopathic behavior profile proposed in the 1960s^9^.

Subsequently, the Type-D (distressed) personality emerged as a significant factor influencing adjustment to and prognosis in cardiovascular diseases [10-12]. Type-D is defined as a joint tendency towards negative affectivity (NA) and social inhibition in interpersonal relationships (SI), similar to neuroticism and introversion, respectively. Type-D NA is the tendency to experience negative emotions over time and in diverse life situations, and Type-D SI is the tendency to inhibit self-expression in social interactions. Studies of Type-D suggest it as a vulnerability factor in people with cardiovascular conditions and also other medical conditions [13], as well as in the general population [14]. A few studies have investigated Type-D personality among cancer patients as a possible predictor of psychosocial disorders, although accumulating data suggest that 25-30% of cancer patients meet the criteria for a range of formal psychiatric diagnoses, including depression, anxiety, and post-traumatic stress disorders and/or symptoms [15, 16].

The NA component of Type- D personality has been associated with an increased risk for all-cause mortality among patients with colorectal cancer, although this adverse effect was limited only to men aged over 70 years [17]. This probably reflects the interaction between high NA and poor health behaviors (*e.g*., inactivity) that, in turn, are also independently associated with poor quality of life and psychological distress [18]. Another study reported a 56% prevalence of Type-D personality in ovarian cancer patients [19], which was associated with greater symptom reporting and lower quality of life. Also, Type-D personality was the only independent predictor of a low level of perceived social support among ovarian cancer patients [20].

Similar findings were obtained in other studies associating Type-D personality with QoL, mental health status, comorbidity burden and health care, as well as illness perception in patients affected by gastric and colorectal cancer [21-26]. More recently, Lv *et al*. [27] found that, amongst lung cancer patients, type D personality significantly and directly predicted the level of psychological distress (63.7% of participants), together with other factors, such as symptom burden, social support, and intrusive thoughts.

Regarding breast cancer, only two cross-sectional studies are available. In an Italian report examining the role of serotonin transporter gene-linked polymorphic region (5-HTTLPR) in increasing the risk of depression, no association was found with Type D personality [28]. In a further cross-sectional French study exploring Type D personality, higher scores were found among breast cancer patients than individuals affected by acute coronary syndrome [29].

To our knowledge, no study has been prospectively conducted to explore the role of Type D personality in molding psychosocial distress conditions in women affected by breast cancer. Therefore, we hypothesized that a trait personality construct, such as Type D, could be significantly related to state conditions, such as distress, anxiety, depression, post-traumatic stress symptoms and maladaptive coping over time. Therefore, the aims of this study were to examine the prevalence and the stability of Type D personality and explore the association between Type-D personality and the above-mentioned clinically-significant psychosocial stress conditions.

## 2 METHODS

### 2.1 Participants

A convenience sample of breast cancer patients was enrolled at the out-patient and day-hospital clinics of the Unit of Medical Oncology, University S. Anna Hospital in Ferrara, North-Eastern Italy. Criteria for recruitment were: (i) a diagnosis of cancer within 6 months; (ii) a Karnofsky Performance Status scale >80; (iii) no cognitive deficits or CNS compromise at clinical evaluation; (iv) age 18-70 years. Each patient completed a comprehensive psychosocial assessment at recruitment within 6 months from diagnosis (baseline, T0) and six months after the first assessment (follow up, T1). All the patients were informed about the aims of the study and their written consent for participation has been obtained. The study followed the regulations and ethics of the Committee for the Protection of Persons as adopted by the Local Health Trust (Azienda Sanitaria Locale di Ferrara, Ferrara, Italy) and approved by the University of Ferrara, and thus conducted accordingly.

Each patient was individually administered a short clinical interview in which the presence of possible psychopathological problems in the past (psychiatric history: yes/no) and stressful life events before diagnosis (yes/no) were assessed. A booklet of self-report psychometric instruments was also given. The sociodemographic and clinical (Karnofsky score, chemotherapy and other cancer treatment) data were extrapolated through the patients’ charts.

### 2.2 Psychosocial Assessments

#### 2.2.1 Type-D Personality

Characteristics of personality were assessed by using the 14-item Type-D (distressed) personality Scale-14 (DS14) [30]. This self-rating scale has been widely used in studies involving cardiovascular patients. In agreement with its theoretical framework, it examines the typical traits of the construct, namely NA (*i.e*., the tendency to experience negative emotions across times and situations) (7 items, α= 0.88, *e.g*. “I often make a fuss about unimportant things”; “I am often in a bad mood”; “I often find myself worrying about something”) and SI (*i.e*., the tendency to inhibit the expression of emotions and behavior in social interactions) (7 items, α= 0.86, *e.g*., “I often feel inhibited in social interactions”; “When socializing, I don’t find the right things to talk about”; “I would rather keep other people at a distance”) on a 5-point Likert scale (from 0 to 4). A cut-off score ≥10 on both scales indicates the presence of Type D personality.

#### 2.2.2 General Distress, Anxiety, Depression and Post-traumatic Stress

General distress was evaluated through the Distress Thermometer (DT) and the BSI-18 Global Stress Index. The DT, developed by the Distress Management Guidelines Panel within the National Comprehensive Cancer Network [31, 32], is a visual analog tool asking the respondent to rate his/her level of distress in the past week on a scale from 0 (no distress) to 10 (extreme distress). It has been widely used in cancer settings [33]. The BSI-18 Global Stress Index was obtained by summing the response to the 18 items of the Brief Symptom Inventory-18 [34], each of which is rated on a 5-point Likert scale (from 0 = not at all to 4 = extremely) reflecting the last 7 days. Score total ranged from 0-72, with higher scores reflecting greater stress-related symptoms. The scale has been widely used in cancer settings [35].

From the BSI-18, the Anxiety subscale (BSI-ANX), consisting of 6 items (α=0.92), was extrapolated to assess anxiety. In order to find clinically significant cases on the BSI-ANX, the recommended case-rule system (conversion of the raw score in standardized T scores, cases =T ≥ 63) was used, as done in other studies [36].

Depression was measured by the 7-item Depression subscale of the Hospital Anxiety-Depression Scale (HAD-D) [37] (0-3 Likert scale: range score 0-21; Cronbach’s α=0.84), a widely used scale among medically ill patients, including cancer patients. As recommended [38, 39] and confirmed by several studies [40, 41], a cut-off ≥11 was considered for “caseness” of depression.

Post-traumatic stress was evaluated through the 15-item Impact of Event Scale (IES) [42], which has been widely used among the medically ill patients, including cancer patients (e.g [43, 44].) to document the emotional impact of events, such as cancer and trauma. The IES uses a 4-point Likert scale to score the frequency of intrusive cognitions and behaviour (Intrusion subscale, 7 items; range score 0–35; in this study α=0.82) and avoidant cognitions and behaviour (Avoidance subscale, 8 items; range score 0–40, α=0.79). Scores are summed to a total IES Total score (range score of 0–75, α=0.87). A cut-off ≥35 indicated high sensitivity (0.89) and specificity (0.94) for PTSD in a validation study involving a formal psychiatric structured interview of a large population of Danish breast cancer patients [45]; it was used here for discriminating PTSD “cases” from “non-cases”.

#### 2.2.3 Coping

Coping was measured using two main sub-factors of the Mini-Mental Adjustment to Cancer (Mini-MAC) scale [46], namely Anxious Preoccupation (Mini-MAC AP) and Hopelessness (Mini-MAC H). Both sub-scales consist of 8 items scored on a 1–4 Likert scale measuring the tendency to feel worried and preoccupied about cancer (AP, α= 0.89) and the tendency to adopt a pessimistic and despairing attitude towards it (H, α= 0.91). All the scales were used in their validated Italian versions [47-51].

### 2.3 Statistical Analysis

Principal component analysis (Varimax rotation) was used to explore the factor structure of the Type-D measure. Descriptive statistics, T-test, chi-square test and Pearson *r* correlation tests and analysis of variance were next used as appropriate to examine correlations and differences between groups. Regression analysis was used to explore the variables related to anxiety, depression, PTSS, and maladaptive coping both on T0 and T1. All analyses were conducted using SPSS 21.0 (IBM corporation), with the level of statistical significance set at p<0.05.

## 3 RESULTS

### 3.1 Sample Characteristics

A total of 159 patients meeting the inclusion criteria were approached during the recruitment period; eight declined participation for several reasons (five for no interest, two had other commitments or lack of time, and one for health reasons) and four had missing measures not allowing to evaluate the tests. The final sample was then composed of 145 subjects (mean age 55.8±8.9), whose socio-demographic and clinical characteristics are reported in Table **1**.

### 3.2 Type D Personality

Principal component analysis (Varimax rotation with Kaiser normalization; Kaiser–Meyer–Olkin measure of sample adequacy = 0.84, p=0.001) identified the same two factors of the original version, plus a third factor consisting of only two items, which explained in total 64.6% of the variance. The first factor consisted of the same 7 items as in the original SI component and explained 42.5% of the variance; the second factor consisted of 5 of the 7 items of the original NA dimension, explaining 15% of the variance; the last two items related to worry (Item 2: I often make a fuss about unimportant things; item 12: I often find myself worrying about something) loaded both on NA but more significantly as a third factor which explained a further 7% of the variance. Given the low number of items in this factor and the loading of these two items close to the NA dimension, we decided to examine the prevalence of the of Type D personality by using the recommended rule-system (both NA and SI ≥10). The prevalence of Type-D was 25.8% (n=37). Good test-retest reliability was seen indicating stability over time of Type D personality, with a significant correlation between scores on the single factors and Type-D total at T0 and T1 (SI r =.71, p < 0.01; NA r=.61, p<0.01; Type-D Total r=.82, p<0.01). Also, the mean scores of the Type D were not significantly different on T0 and T1 (NA 11.1 ± 6.84 *vs*. 10.53 ± 6.9, t =1.5, p=ns; SI 7.76 ± 6.73 *vs*. 7.82 ± 6.86, t=0.18, p=ns; Type D Total 18.83±11.63 *vs*. 0 18.34 ±: 11.63, t=0.83, p=ns.). Type D was not related to age, marital status (F=1.6, p=0.09) and medical variables (*i.e*., stage of cancer, F=0.15, p=0.96), nor occurrence of life events before diagnosis (F=0.23, p=0.63). Patients reporting a lifetime history of psychological disorders were more likely to have higher scores on Type D (F=7.33, p< 0.01; Type D cases 32% *vs*. 19%, p < 0.05).

### 3.3 Changes in Psychosocial Distress Over Time and Correlation

There were changes observed in the several psychosocial dimensions over time, with a reduction in the scores of all scales, except for HADS-D Fig. (**1**) and (Table **S1**). Table **2** shows the correlation between the variables. Type D personality was significantly associated with all psychosocial variables at both T0 and T1 (r range from .31 to .53, p< 0.01). Among the single components, NA was more significantly related to the independent variables (r range from .37 to .62, p < 0.01) in comparison to SI that showed slightly lower correlations which remained significant (r range from .18 to .32, p < 0.05) with the exception of IES-Avoidance. These findings were confirmed when the prevalence of Type-D personality was examined among cases of depression, anxiety, post-traumatic stress and general distress (data available on request from the authors).

### 3.4 Regression Analyses with Type-D

A first multiple regression analysis was performed in which personality (*i.e*., Type-D Total, Type-D SI, Type-D NA), social (*i.e*., age, history of psychiatric disorders; life events), and medical variables (*i.e*. stage of cancer, treatment) were regressed on each of the psychosocial stress variables (dependent variables), namely DT, BSI-GSI, HAD-Depression, BSI-ANX, IES (Avoidance, Intrusion, Total), Mini-Mac H and AP scores, at T0. A second regression analysis was performed with the same psychosocial stress variables (dependent variables) at T1. As general findings, at T0, Type D personality and its SI component were predictors for all the psychosocial explored dimensions, with other variables being also predictors for some conditions (*i.e*., previous history of psychopathology for BSI-GSI, BSI-Anxiety, and HADS-D; stressful events for BSI-GSI, IES Total, IES Intrusion, and Mini-MAC Hopelessness). At T1, Type-D personality and its Type-D SI and NA components were predictors for all the psychosocial explored dimensions and a few others in some conditions (*i.e*., stressful events for IES Total, Intrusion and Mini-MAC Hopelessness; the stage for DT, HAD-D, Mini-MAC Hopelessness). The details are shown in Table **3**.

## 4 DISCUSSION

This study aimed at examining the relationship between Type D (distressed) personality and the psychological response to breast cancer at two different points of time.

A first result was that the prevalence of Type D personality (as characterized by both negative affectivity and social inhibition) was lower (about 25%) than that reported in some studies carried out in cancer settings. The Type D personality became stable over time, confirming the reliability of the construct, as indicated by previous results of the analysis of Type D in medically ill patients. In our study, patients with a history of psychological disorders were at a higher risk of being “caseness” for Type D, while this personality construct was not related to stressful events, stage of cancer, Karnofsky score, cancer treatment, or age. This finding could be interpreted as the fact that Type-D personality may make, or reflect, people more prone to develop psychological disorders, as already shown in other prospective studies of the general populations, akin to neuroticism. It is in fact quite understandable that personality traits oriented to negative affectivity and social inhibition prevent individuals from responding to stressful events in a constructive and adaptive way, exposing them to a risk of developing psychological distress conditions.

A second related result was that Type-D was found to be significantly associated with all the dimensions of psychosocial maladjustment as anxiety, depression, post-traumatic stress symptoms and general distress, both within six months after diagnosis (T0) and, prospectively, six months later (T1). Also, Type-D personality was associated with maladaptive coping styles, namely the tendency to feel worried and preoccupied about cancer (anxious preoccupation) and the tendency to adopt a pessimistic and despairing attitude about it (hopelessness). Of the two components, negative affectivity, in terms of the tendency to experience negative emotions over time and in diverse life situations, was particularly associated with psychosocial distress variables indicating the concomitant influence of a negative affectivity proneness and the parallel negative reaction to the cancer experience. Also, social inhibition, as the tendency to inhibit self-expression in social interactions, had its association with the same psychosocial dimensions, with the exception of the avoidance component of post-traumatic stress. Interestingly, however, when examined in regression analysis, social inhibition emerged as a more significant factor, and for some dimensions, occurrence of stressful life events and previous psychological disorders in the past, in predicting psychosocial distress and maladaptive coping both on the first and the second assessments. These findings underline that not only the component of pessimism and negative affectivity (with its possible overlap with neuroticism) tend to be part of a construct that is associated with the onset of psychological disorders in several domains, including anxiety, depression and post-traumatic stress, but the tendency to inhibit oneself in social interaction, and therefore, likely to receive less social support form interpersonal ties is also a significant factor.

Taken together, the findings are in line with the only other study available in cancer patients [26], suggesting that a quick examination of some personality parameters, such as negative affectivity and social inhibition, could help clinicians in identifying patients who can be more vulnerable to both develop different psychological disorders, including depression, anxiety, post-traumatic stress and general distress symptoms, and to use the cognitive and behavioral response to cancer (coping) characterized by maladaptive mechanisms.

## CONCLUSION

There are several limitations of the study. A first is that the sample size does not allow to generalize the results of the study. Secondly, patients were affected by breast cancer; thus, more studies in Italy on patients affected by other types of cancer are necessary. Furthermore, all the data were gathered by using psychometric tools, which although validated may have determined some bias or loss of information in defining the several clinical conditions affecting cancer patients. Third, it is necessary to consider that, although two constructs are part of Type-D, NA is the biggest component of other variables measured in the study (*e.g*., DT, BSI-GSI, HAD-D, IES), with the risk of collinearity that indicates the need for larger samples to better explore the association between the variables and reduce overgeneralization.

In conclusion, the study confirms the findings of other studies involving medically ill patients (especially cardiology), that a personality construct, such as Type-D (distressed) personality, and its dimension of negative affective and social inhibition, are related to symptoms of psychological distress and maladaptive coping. Information on cancer settings where Type-D has been explored mostly in terms of quality of life and progression of the disease.

## Figures and Tables

**Fig. (1):**
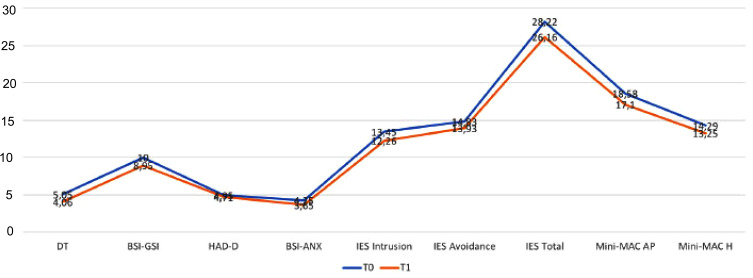
Scores of psychosocial variables at the 2-time point assesment

**Table 1:** Sociodemographic and clinical characteristics of the sample (n=145).

Variables	N (%)
*Age* (yrs) mean = 55.87 ± 8.98 (Range: 24-70) young adults (18-39 yrs) adults (40-59 yrs) seniors (≥60 yrs)	6 (4.2%) 81 (55.8%) 58 (40%)
*Education* (yrs) mean 9.76 ± 4.34 (Range 5-18) elementary school (5 yrs) middle school (13 yrs) high school (13 yrs) university (> 13 yrs)	35 (24.1%) 50 (34.5%) 44 (30.3%) 16 (11.1%)
*Marital status* Never-married Separated/divorced Married Widows	11 (7.6%) 15 (10.3%) 110 (75.8%) 9 (6.2%)
*Occupation* Employed Unemployed Housewife Retired	65 (44.8%) 7 (4.8%) 29 (20%) 44 (30.3%)
*Past psychological disorders* Yes No	50 (34.5%) 95 (65.5%)
*Stage* Local disease Loco-regional	123 (84.8%) 22 (15.2%)
Surgery * Local treatment Non- conservative	102 (64.1%) 43 (29.6%)
*Systemic Therapies* No Therapy Chemotherapy Combined therapy (chemio +/-hormone, +/- biologic therapy, +/- radiotherapy)	70 (48.3%) 54 (37.2%) 21 (14.5%)

*Local treatment: Conservative surgery (*i.e*., quadrantectomy, lumpectomy and tumorectomy); Non-conservative surgery (mastectomy).

**Table 2:** Correlation between Type D and psychosocial variables at T0 and T1.

**Psychosocail Variables**	**Type D**			**Negative Affectivity**		**Social Inhibition**	
	T0		T1	T0	T1	T0	T1
DT	.48		.40	.50	.41	.31	.28
BSI-18 GSI	.54		.49	.61	.54	.32	.26
HADS-D	.54		.47	.56	.46	.35	.33
BSI-18 Anxiety	.51		.47	.62	.53	.25	.28
IES Total	.41		.39	.52	.46	.17	.23
Intrusion	.38		.42	.47	.47	.18	.25
Avoidance	.35		.31	.47	.37	.13*	.16*
Mini-MAC Anxious Preoccupation	.54		.54	.61	.54	.31	.37
Mini-MAC Hopelessness	.54		.48	.56	.51	.37	.32

DT = Distress Thermometer; BSI-GSI = Brief Symptom Inventory- Global Stress index HAD-D: Hospital Anxiety and Depression – Depression subscale; IES = Impact of Event scale; Mini-MAC= Mini-Mental Adjustment to Cancer scale. All p < 0.05 except * = ns.

**Table 3:** Regression analysis at T0 and T1

**-**	**Predictors**	**Variance explained**	**Beta**	**F**	**p**
***T0***					
BSI-18 GSI	History psychopathology Stressful events Type D SI Type D Total	38%	.15 .12 .45. .91	23.5	0.001
DT	Type D Total Type D SI	26%	.15 .63 .32	25.9	0.001
IES Total	Stressful events Type D SI Type D Total	30%	.15 .61 .94	16.15	0.001
Intrusion	Stressful events Type D Total Type D SI	29%	.19 .86 .52	15.8	0.001
Avoidance	Type D SI Type D Total	21%	.61 .86	19.76	0.001
HAD-Depression	History psychopathology Type D SI Type D Total	34%	.14 .35 .81	25.4.	0.001
BSI-18 Anxiety	History psychopathology Type D SI Type D Total	38%	.17 .61 .98	30.7.	0.001
Mini-MAC Hopelessness	Stressful events Stage Type D SI Type D Total	34%	.12 .11 .35 .84	19.81	0.001
Mini-MAC Anxious Preoccupation	Type D SI Type D Total	37%	.56 1.02	43.27	0.001
***T1***					
BSI-18 GSI	Type D Total Type D SI	25%	.64 2.1	24.31	0.001
DT	Stage Type D Total Type D SI	22%	.15 .63 .32	14.33	0.001
IES Total	Stressful events Type D SI Type D Total	20%	.13 .72 .37	12.76	0.001
Intrusion	Stressful events Type D Total	19%	.20 .43	19.95	0.001
Avoidance	Type D Total	8%	.29	12.9	0.001
HAD-Depression	Stage Type D Total Type D NA Type D SI	27%	.29 .51 .21 .28	14.45	0.001
BSI-18 Anxiety	Type D Total Type D SI	23%	.63 .22	22.9	0.001
Mini-MAC Hopelessness	Stage Stressful events Type D Total Type D NA Type D SI	34%	.21 .12 .96 .23 .45	15.87	0.001
Mini-MAC Anxious Preoccupation	Type D Total	28%	.54	57.96	0.001

BSI-18 GSI = Brief Symptom Inventory 18 - Global Stress index; DT = Distress Thermometer; HAD-S: Hospital Anxiety and Depression Scale; IES = Impact of Event; SI = Type D Social Inhibition scale; NA: Type D Negative Affectivity; Mini-MAC= Mini-Mental Adjustment to Cancer scale.

## Data Availability

Not applicable.
