# Supplementary material for: Association between Type-D Personality and Affective (Anxiety, Depression, Post-traumatic Stress) Symptoms and Maladaptive Coping in Breast Cancer Patients: A Longitudinal Study
Source: Clin Pract Epidemiol Ment Health. 2021 Dec 31;17(Supp-1):271–9. doi: 10.2174/1745017902117010271 (PMC8985468; doi:10.2174/1745017902117010271)
Supplement: Supplementary file 1 — Supplementary material is available on the publisher’s website along with the published article. [file CPEMH-17-271_SD1.pdf]

1745-0179/21 Send Orders for Reprints to reprints@benthamscience.net

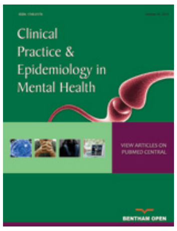

Clinical Practice & Epidemiology in Mental Health

Content list available at: https://clinical-practice-and-epidemiology-in-mental-health.com

Supplementary Material

Association between Type-D Personality and Affective (Anxiety, Depression, Post-traumatic Stress) Symptoms and Maladaptive Coping in Breast Cancer Patients: A Longitudinal Study

Luigi Grassi[1,2,*], Rosangela Caruso[1,2], Martino Belvederi Murri[1,2], Richard Fielding[3], Wendy Lam[3], Silvana Sabato[1], Silvia De Padova[4], Maria Giulia Nanni[1,2], Tatiana Bertelli[4] and Luigi Zerbinati[1,2]

[1]*Department of Neuroscience and Rehabilitation, Institute of Psychiatry, University of Ferrara, Ferrara, Italy*
[2]*University Hospital Psychiatry Unit, University S. Anna Hospital and Local Health Trust, Ferrara, Italy*
[3]*Centre for Psycho-Oncological Research and Training, School of Public Health, The University of Hong Kong, Hong Kong School of Public Health, The University of Hong Kong, Pok Fu Lam, Hong Kong; Hong Kong Special Administrative Region, People's Republic of China*
[4]*Psycho-Oncology Unit, IRCCS Istituto Romagnolo per lo Studio dei Tumori (IRST) "Dino Amadori", Meldola, Italy*

Table S1. Differences in the scores of psychosocial variables at the 2-time point assessment.

Assessment	T0	T1	t	p
Distress Thermometer	5.05 ± 2.74	4.06 ± 2.81	4.12	0.001
BSI-18 General Stress Index	10.07 ± 10.95	8.95 ± 11.05	1.91	0.05
HAD-D	4.95 ± 4.46	4.71 ± 4.32	0.77	ns
IES-Intrusion	13.45 ± 5.14	12.26 ± 4.94	3.55	0.01
IES-Avoidance	14.83 ± 5.29	13.93 ± 5.48	2.21	0.03
IES-Total	28.22 ±9.53	26.16 ± 9.62	3.18	0.01
BSI-18 Anxiety	4.25 ± 4.5	3.65 ± 4.6	2.32	0.02
Mini-MAC Anxious Preoccupation	18.58 ± 5.46	17.1 ± 5.41	4.74	0.01
Mini-MAC Hopelessness	14.29 ± 5.26	13.25 ± 4.87	3.36	0.01

BSI = Brief Symptom Inventory; HAD-D: Hospital Anxiety and Depression – Depression subscale; IES = Impact of Event; Mini-MAC= Mini-Mental Adjustment to Cancer Scale.

© 2021 Grassi *et al*.

This is an open access article distributed under the terms of the Creative Commons Attribution 4.0 International Public License (CC-BY 4.0), a copy of which is available at: https://creativecommons.org/licenses/by/4.0/legalcode. This license permits unrestricted use, distribution, and reproduction in any medium, provided the original author and source are credited.

DOI: 10.2174/1745017902117010271, 2021, *17*, (Suppl-1, M4) i-i
